# Editorial: The wide spectrum of ventricular arrhythmias: from out-of-hospital cardiac arrest to advanced in-hospital treatment

**DOI:** 10.3389/fcvm.2024.1361013

**Published:** 2024-02-02

**Authors:** Enrico Baldi, Hanno L. Tan, Veronica Dusi, Roberto Rordorf, Alessandro Zorzi, Simone Savastano

**Affiliations:** ^1^ Arrhythmias and Electrophysiology Unit, Division of Cardiology, Fondazione IRCCS Policlinico San Matteo, Pavia, Italy; ^2^ Cardiac Arrest and Resuscitation Science Research Team (RESTART), Fondazione IRCCS Policlinico San Matteo, Pavia, Italy; ^3^ Department of Clinical and Experimental Cardiology, Heart Center, Amsterdam Cardiovascular Sciences, Amsterdam UMC Location AMC, University of Amsterdam, Amsterdam, Netherlands; ^4^ Netherlands Heart Institute, Utrecht, Netherlands; ^5^ Cardiology, Department of Medical Sciences, University of Turin, Torino, Italy; ^6^ Division of Cardiology, Cardiovascular and Thoracic Department, “Città della Salute e della Scienza” Hospital, Torino, Italy; ^7^ Inherited Cardiomyopathy and Sports Cardiology Unit, Department of Cardiac Thoracic and Vascular Science and Public Health, University of Padova, Padova, Italy

**Keywords:** ventricular arrhythmias, non-invasive ablations, out-of-hospital cardiac arrest patients, arrhythmic risk stratification, cardiomyopathies, inherited cardiac arrhythmias

**Editorial on the Research Topic** The wide spectrum of ventricular arrhythmias: from out-of-hospital cardiac arrest to advanced in-hospital treatment

## Introduction

Ventricular arrhythmias (VAs) are one of the most life-threatening acute clinical conditions (1) and may be responsible of a different clinical scenarios from out-of-hospital cardiac arrest (OHCA), whose outcome is bounded to the speed and effectiveness of rescue system, comprising both citizen-rescuers and emergency medical system (EMS) (2), to isolated or recurrent relapses in patients with an implantable cardioverter-defibrillator (ICD) or hospitalized for different medical conditions. Their prediction and treatment are some of the most fascinating fields for physicians involved in the management of patients with VAs. Despite a significant progress made in recent years, many gaps in knowledge exist. To refine our capability of arrhythmic risk stratification by advanced imaging tools and electrophysiological tests is a challenge for clinicians, both in patients with ischemic and non-ischemic cardiomyopathies and in those with inherited cardiac arrhythmias. Finally, the acute and chronic treatment of VAs, starting from the pre-hospital setting throughout the in-hospital phase and after discharge regardless of the clinical presentation, is crucial for survival. New techniques emerged over the last years that let us imagine even better outcomes in the future. The goal of the Research Topic was to expand the knowledge regarding all the aspects related to VAs.

### Arrhythmic risk stratification

Russo et al. reported an association, in a patient with a family history of sudden death and dilated cardiomyopathy, and inducibility of polymorphic ventricular tachycardia at electrophysiological study, of a drug-induced type 1 Brugada pattern and a Ser573Leu missense variant on the Lamin A/C (*LMNA*) gene. The authors suggested that the Brugada pattern might be part the cardiolaminopathies spectrum, proposing *LMNA* genetic testing for all the patients with drug-induced type 1 Brugada pattern.

Moreover, Tsartsalis et al. carried out a review of fifty-five meta-analyses of observational and randomized controlled trials to explore the modifiable risk and protective factors of sudden cardiac death (SCD). The authors concluded that lifestyle risk factors (physical activity, smoking), comorbidities as diabetes, and electrocardiographic features like early repolarization constitute modifiable risk factors of SCD. Alternatively, the use of mineralocorticoid receptor antagonists (MRAs), beta-blockers, and sodium-glucose cotransporter-2 (SGLT-2) inhibitors are protective factors. This evidence opens the possibility for future investigations targeted in specific populations aimed to reduce the SCD burden.

### Improving the “chain of survival”

In a survey conducted by Kovoor et al. among the Australian public, a newly designed yellow-red signage for AEDs and cabinets was preferred by 73.0% and 88%, respectively, over the green-white counterparts. The authors therefore proposed to standardize yellow-red signage of AED and cabinet as it is easier to identify over the green-white one.

Furthermore, Nielsen et al. demonstrated a four-fold increased odds ratio for bystander CPR and a three-fold increased odds ratio for bystander defibrillation when volunteer responders accepted the alarm and arrived before EMS. This study, which used data from 1877 OHCAs with volunteer responders activation in Denmark, stresses the importance of the activation of persons trained in CPR/AED, which can also improve patients' survival (3).

### Advanced pre-hospital treatment strategies

In an international multicenter observational study, Gentile et al. evaluated the value of Amplitude Spectral Area (AMSA) of VF of 2077 shocks. This study, called MOSAIC study, demonstrated that the administration of amiodarone was independently associated with the probability of recording lower values of AMSA. Moreover, the authors highlighted the fact that the predictive value of AMSA for shock success is significantly lower, but still statistically significant, in patients who have received amiodarone during cardiac arrest. This topic seems to be of great clinical importance considering that AMSA is an emerging indicator that might guide defibrillation and resuscitation efforts (4). Taking into account the results of the MOSAIC study, further randomized study are needed to clarify the effect of amiodarone on AMSA.

### Improving hospital treatment and long-term management

The features of patients who underwent subcutaneous implantable cardiac defibrillator (S-ICD) implantation in the clinical practice of a single center, as well as the ICD-related complications and the inappropriate therapies during follow-up were described by Russo et al. The authors highlighted that the choice to implant an S-ICD was mainly driven by the younger age and by the presence of a channelopathy; conversely ischemic cardiomyopathy reduces the probability to use this technology. As in previous reports (5), no differences in inappropriate ICD therapies were shown among S-ICD vs. transvenous ICDs (TV-ICD) and a lower rate of infectious and non-infectious complications leading to surgical revision or extraction were observed in S-ICD recipients. Also with regard to ICD patients, Regoli et al. carried out a review on the management of hemodynamically stable, incessant wide QRS complex tachycardia in patients who already received an ICD, which represents a challenging situation. The authors proposed an approach based on four different phases to deal with these patients: hemodynamic status assessment; preparation for therapy and removal of potential triggers; therapy administration including anti-arrhythmic drugs, device reprogramming and acute neuromodulation, which is promising in treating this type of patients (6); hospitalization and prevention of arrhythmic relapses.

Concerning patients with drug-refractory electrical storm (ES), Cojocaru et al. highlighted that patients with non-revascularized chronic total occlusions (NR-CTO) demonstrated a higher ratio of border-zone to total scar area compared to patients without NR-CTO. Moreover, NR-CTO seemed to be associated with worse acute procedural results and may as well impact long-term outcomes.

Moving to non-invasive VAs ablation, Whitaker et al. carried out a review concerning cardiac stereotactic body radiation therapy (cSBRT) for VAs refractory to medical therapy and catheter ablation. The authors stressed how this technique is promising to treat VAs, but also that many aspects related to this technique are currently unknown, especially concerning the radiobiology of the anti-arrhythmic effect, representing an exciting opportunity for future research.

In conclusion, VAs represent a complex challenge which requires the involvement of multiple actors, from the community to expert in hospitals, and the use of multiple techniques to increase the patients' chances of survival.

## Conclusion and future perspective

VAs' treatment relies on a multifaceted approach including risk stratification, out and inside hospital treatment and long-term management. Emerging techniques will help clinicians in the future to refine risk and improve treatment to increase outcome, which represent the final aim of research.
